# Demodex-induced Lupus miliaris disseminatus faciei: A case report

**DOI:** 10.1097/MD.0000000000021112

**Published:** 2020-07-02

**Authors:** Yang Luo, Lan-Xi Wu, Jian-Hong Zhang, Nan Zhou, Xiu-Li Luan

**Affiliations:** Department of Dermatology, The 940th Hospital of Joint Logistics Support Force of the Chinese People's Liberation Army, Lanzhou, Gansu Province, China.

**Keywords:** caseation necrosis, Demodex, lupus miliaris disseminatus faciei, ornidazole tablets, recombinant bovine basic fibroblast growth factor gel

## Abstract

**Rationale:**

Lupus miliaris disseminatus faciei (LMDF) is an inflammatory granulomatous skin disease without a clear etiology that frequently involves the middle area of the face and the upper eyelids. Pathological features of the disease include caseation necrosis and epithelioid granuloma. Consensus treatment for LMDF is currently unavailable.

**Patient concerns:**

A 47-year-old Chinese female patient who presented with facial pruritic, erythematous papules 8 months before this study. She was diagnosed with skin tuberculosis at another hospital and given antituberculosis medication. However, the treatment was not efficacious.

**Diagnoses:**

In this study, the diagnosis of Demodex-induced LMDF was made by a dermatologist according to physical examination, skin biopsy pathology, and microscopic examination.

**Interventions:**

The patient was given ornidazole tablets (500 mg twice a day) and recombinant bovine basic fibroblast growth factor gel (0.2 g/cm^2^ twice a day) for an 8-week period.

**Outcomes:**

Eight weeks after the treatment, the facial erythematous papules were improved, and no new skin lesions were observed. The patient showed no signs of recurrence during the 6-month follow-up.

**Lessons subsections:**

This case showed that ornidazole combined with recombinant bovine basic fibroblast growth factor gel might be useful in treatment of Demodex-induced LMDF. In addition, the results suggested that pathological caseation necrosis was caused by a series of inflammatory and immune responses to Demodex infection.

## 1 Introduction

Lupus miliaris disseminatus faciei (LMDF) is a chronic inflammatory skin disease of unknown etiology, and it is characterized by red-brown to yellow-brown dome-shaped papules over the middle area of the face and upper eyelids.^[1]^ The pathogenesis of LMDF is not clear. *Propionibacterium* and *Mycobacterium tuberculosis* are reported to be the pathogen that may cause LMDF, while later *Mycobacterium tuberculosis* was excluded.^[2,3]^ Histological features of LMDF include an epithelioid cell granuloma accompanied by caseation necrosis. A definite treatment plan for LMDF is currently unavailable.^[4]^ Here, we report the case of a patient with LMDF cured by treatment with ornidazole, which fights against Demodex, suggesting Demodex mites may be the cause of LMDF.

## 2 Case report

**Figure 1:**
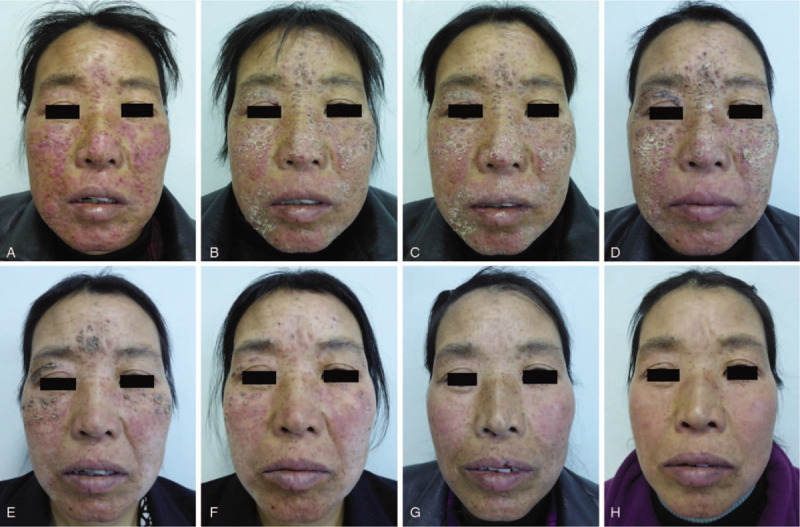
Facial presentation. A, The lesions were scattered erythematous papules with a diameter of 0.3 to 1 cm, and they were symmetrically distributed on the nose, cheeks, forehead, and mandible (before treatment). B–F, The lesions were progressively alleviated at 1 week (B), 2 weeks (C), 3 weeks (D), 4 weeks (E), and 5 weeks (F) after treatment with azole tablets combined with recombinant bovine basic fibroblast growth factor gel. G, Nodules and papules subsided with local residual pigmentation after 8 weeks of treatment. H, No new lesions at the 6-month follow-up.

A 47-year-old Chinese female patient presented with facial pruritic, erythematous papules since the last 8 months. The lesions were symmetrically distributed over the nose, cheeks, forehead, and mandible. They were scattered erythematous papules with a diameter of 0.3 to 1 cm, and a few papulopustules were observed. Radial stripes were observed around the mouth. The patient felt that the facial skin was dry, tight, hot, and itchy, and it was difficult to open the mouth. No obvious abnormalities were found in other skin mucosal tissues (Fig. 1A). The patient did not have any symptoms, such as fever, night sweats, and cough. The patient was physically healthy. She denied any history of tuberculosis and did not have any obvious abnormalities in the other systems. She was diagnosed with skin tuberculosis at another hospital. She was given oral isoniazid tablets (300 mg per day), rifampicin capsules (450 mg per day), pyrazinamide tablets (1.5 g per day), and ethambutol tablets (750 mg per day) for 8 weeks, followed by isoniazid and rifampicin for 16 weeks. However, the treatment was not efficacious, and the skin lesions did not subside and they progressively worsened. Because of nausea, vomiting, and other symptoms after medication, the patient discontinued the drugs for 8 weeks. The skin lesions gradually spread over the entire face.

**Figure 2:**
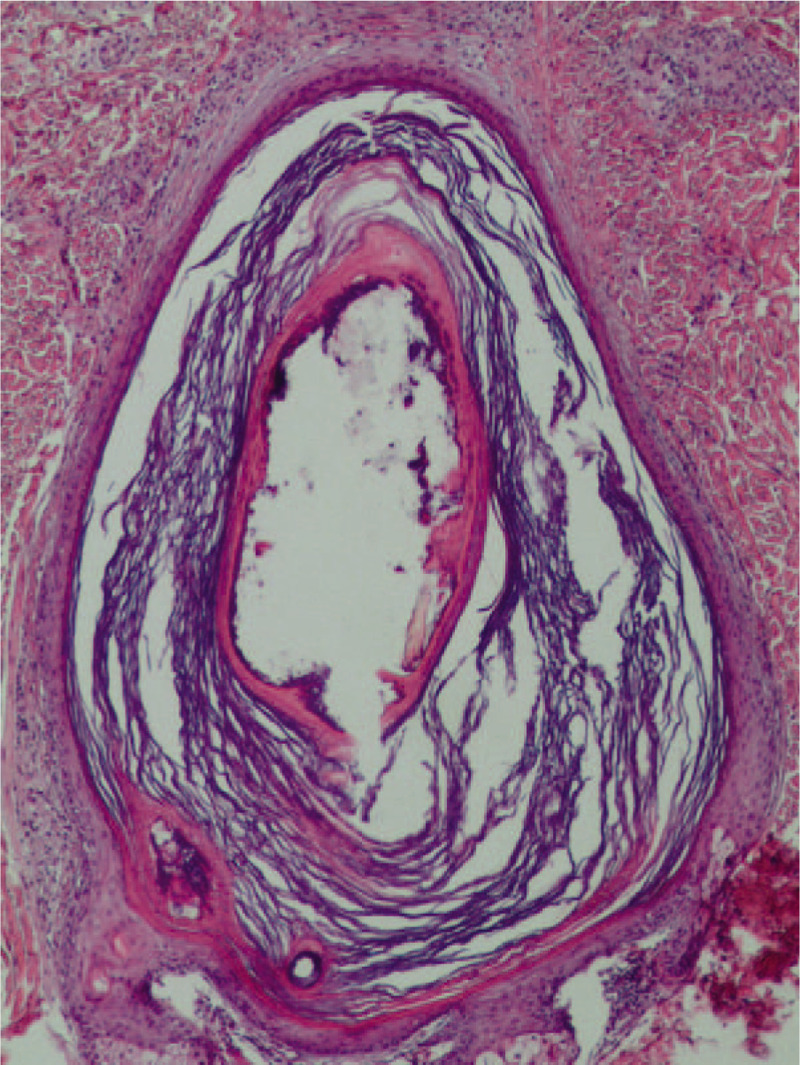
Skin biopsy pathology (hematoxylin-eosin staining). A Demodex mite inside the pilosebaceous unit surrounded by chronic inflammatory tissues, Demodex mite showing mouthparts and body wall structure (×100).

**Figure 3:**
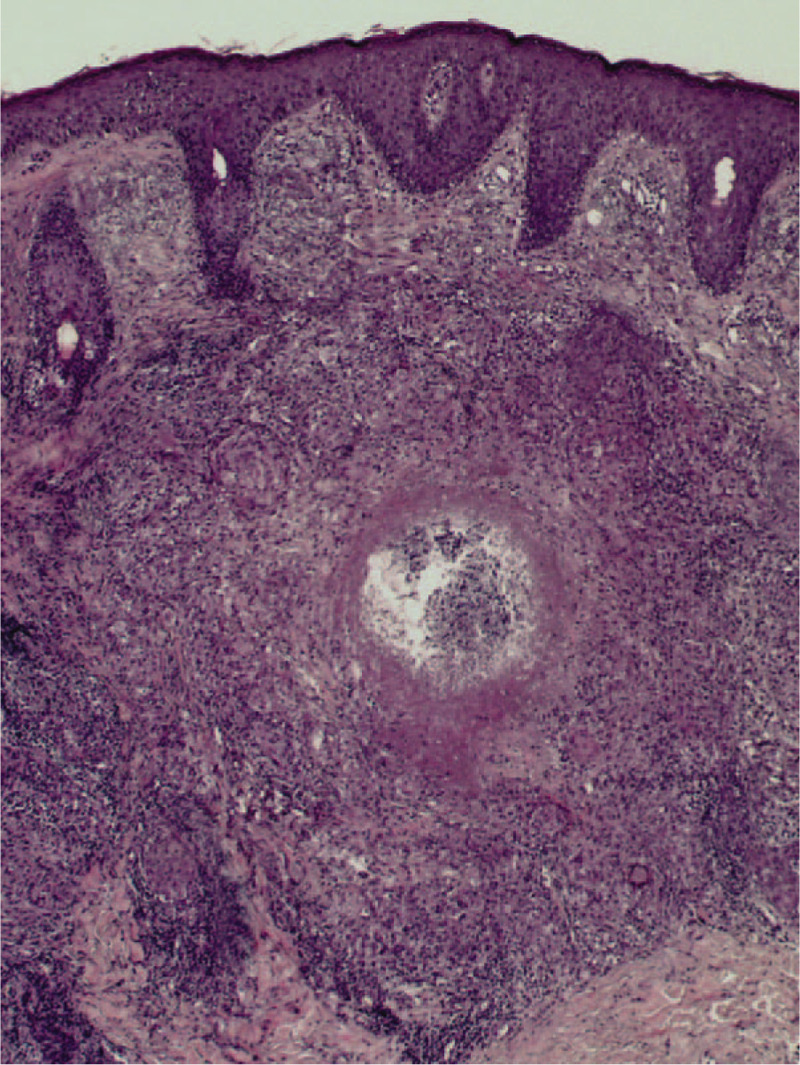
Caseation necrosis in the upper part of the dermis. A tuberculous granuloma comprising epithelioid cells and multinucleated giant cells, surrounded by lymphocytic infiltration and central foci of caseation necrosis (×100).

**Figure 4:**
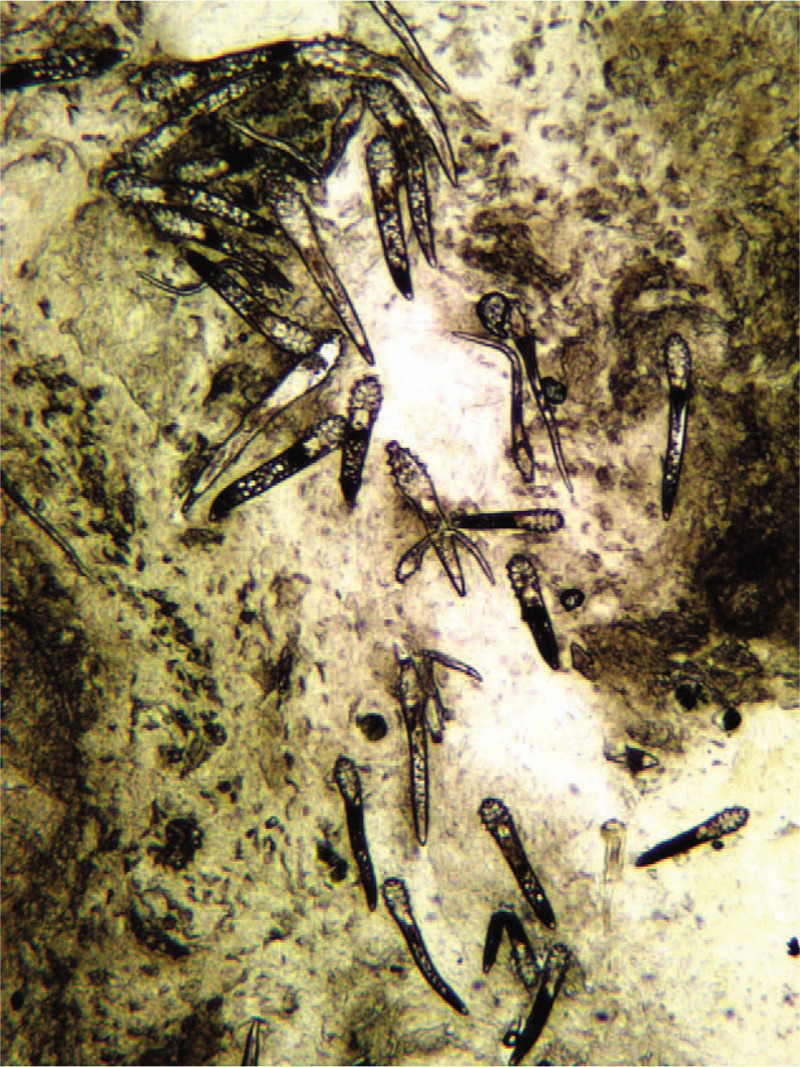
A large number of Demodex mites were observed in the secretion from the pilosebaceous unit over the lesion area in the patient (×40).

A skin biopsy showed the presence of a Demodex mite inside the pilosebaceous unit surrounded by chronic inflammatory tissues in newly developed lesions (Fig. 2). Caseation necrosis in the dermis was observed in mature lesions. Caseation necrosis showed the following 3-layer structure: central foci of caseation necrotic area, a middle layer of tuberculous granuloma formed by epithelioid cells and multinucleated giant cells, and an outer layer of lymphocytic infiltration (Fig. 3), Acid-fast staining was negative. Demodex mites were observed in the secretion of sebaceous glands attached to the hair follicles in lesions (Fig. 4), On the basis of clinical manifestations, laboratory tests, and pathological features, a diagnosis of Demodex-induced LMDF was established. On the basis of our previous study,^[5,6]^ the patient was given ornidazole tablets (500 mg twice a day) to fight against Demodex and recombinant bovine basic fibroblast growth factor gel (0.2 g/cm^2^ twice a day) for the repair of the inflammatory granuloma for an 8-week period. Five weeks after treatment, the lesions gradually improved and the patient reported a reduction in itching (Fig. 1B–F). Eight weeks after the treatment, the facial erythematous papules were improved, and the symptoms of dryness, tightness, burning, and itching were alleviated. No new skin lesions were observed (Fig. 1G). At the 6-month follow-up, the original lesions had completely resolved; there was no facial dryness, tightness, burning, and itching, and no new skin lesions were observed (Fig. 1H).

## 3 Discussion

LMDF is an inflammatory granulomatous skin disease without a clear etiology that frequently involves the middle area of the face and the upper eyelids.^[1]^ The lesions are small, discrete red-yellow, or yellow-brown papules.^[1]^ Pathological features of the disease include caseation necrosis and epithelioid granuloma, which are similar to the pathological features of cutaneous tuberculosis. Cutaneous tuberculosis is a relatively rare manifestation of tuberculosis. Its pathological features include a tuberculous granuloma comprising epithelioid cells and Langhans giant cells surrounded by nodular lymphocytic infiltration, with central foci of caseation necrosis. Several other diseases such as tuberculoid leprosy and granulomatous rosacea (GR) share the same structure of caseation necrosis.^[7,8]^ Thus, LMDF was originally considered to be tuberculoderma. However, *Mycobacterium tuberculosis* was not found in active lesions, and it was eventually determined that LMDF was not related to tuberculosis.^[2]^ This explains why antituberculosis treatment was ineffective in the present case. Several factors, such as Propionibacterium, were hypothesized to be associated with LMDF, but no solid evidence was found to support this association.^[3]^

LMDF has also been considered to be a granulomatous form of rosacea.^[9]^ Studies have shown that Demodex might play an important role in the pathogenesis of rosacea through mechanical stimulation, secondary bacterial infection, and immunopathology, especially in the pathogenesis of papulopustular rosacea and GR.^[10–12]^ GR is an atypical variant of rosacea that is characterized by papular and granulomatous lesions; and on histopathological examination, it shows an epithelioid granuloma.^[13]^ GR always involves the middle area of the face, and lesions are characterized by erythema, telangiectasia, pustules, flushing, and edema,^[14]^ which is different from the clinical manifestations of LMDF.^[15]^ In the present case, caseation necrosis was observed in mature lesions, and Demodex mites were detected in the secretion from lesions. A Demodex mite wrapped in the chronic inflammatory tissue was also observed in newly developed lesions. The present case was diagnosed as LMDF on the basis of the pathological results and clinical manifestations.

The treatment outcome for LMDF is usually unsatisfactory. Tetracycline is a commonly used first-line drug. Other systemic treatments include isotretinoin and dapsone.^[16,17]^ Pathological features in the present case were similar to those of lesions caused by Demodex infection, as reported previously.^[5,6]^ Thus, ornidazole tablets combined with recombinant bovine basic fibroblast growth factor gel were used to fight against Demodex and to repair skin lesions, respectively. The treatment outcome suggested that pathological caseation necrosis may be caused by a series of inflammatory and immune responses to Demodex infection.

In conclusion, this patient with LMDF showed Demodex in newly developed lesions and caseation necrosis in mature lesions. The patient was successfully cured by treatment against Demodex for 8 weeks, and she showed no signs of recurrence during the 6-month follow-up. The skin biopsy pathology, microscopic examination, and treatment outcome suggested that pathological caseation necrosis was caused by a series of inflammatory and immune responses to Demodex infection. However, the pathogenic mechanism of Demodex in LMDF needs to be studied further.

## Author contributions

**Formal analysis:** Yang Luo.

**Investigation:** Yang Luo, Lan-Xi Wu, Jian-Hong Zhang, Nan Zhou.

**Methodology:** Yang Luo, Lan-Xi Wu, Jian-Hong Zhang.

**Project administration:** Yang Luo, Lan-Xi Wu.

**Supervision:** Lan-Xi Wu, Jian-Hong Zhang, Nan Zhou.

**Visualization:** Yang Luo, Xiu-Li Luan.

**Writing – original draft:** Lan-Xi Wu, Jian-Hong Zhang, Nan Zhou.

**Writing – review & editing:** Yang Luo, Xiu-Li Luan.
